# Toward Precise Localization of Abnormal Brain Activity: 1D CNN on Single Voxel fMRI Time-Series

**DOI:** 10.3389/fncom.2022.822237

**Published:** 2022-04-27

**Authors:** Yun-Ying Wu, Yun-Song Hu, Jue Wang, Yu-Feng Zang, Yu Zhang

**Affiliations:** ^1^Center for Cognition and Brain Disorders and the Affiliated Hospital, Hangzhou Normal University, Hangzhou, China; ^2^Zhejiang Key Laboratory for Research in Assessment of Cognitive Impairments, Hangzhou, China; ^3^Institutes of Psychological Sciences, Hangzhou Normal University, Hangzhou, China; ^4^Institute of Sports Medicine and Health, Chengdu Sport University, Chengdu, China; ^5^Transcranial Magnetic Stimulation Center, Deqing Hospital of Hangzhou Normal University, Huzhou, China; ^6^Research Center for Healthcare Data Science, Zhejiang Lab, Hangzhou, China

**Keywords:** fMRI, single-voxel analysis, continuous task states, wavelet transformation, 1D-CNN

## Abstract

Functional magnetic resonance imaging (fMRI) is one of the best techniques for precise localization of abnormal brain activity non-invasively. Machine-learning approaches have been widely used in neuroimaging studies; however, few studies have investigated the single-voxel modeling of fMRI data under cognitive tasks. We proposed a hybrid one-dimensional (1D) convolutional neural network (1D-CNN) based on the temporal dynamics of single-voxel fMRI time-series and successfully differentiated two continuous task states, namely, self-initiated (SI) and visually guided (VG) motor tasks. First, 25 activation peaks were identified from the contrast maps of SI and VG tasks in a blocked design. Then, the fMRI time-series of each peak voxel was transformed into a temporal-frequency domain by using continuous wavelet transform across a broader frequency range (0.003–0.313 Hz, with a step of 0.01 Hz). The transformed time-series was inputted into a 1D-CNN model for the binary classification of SI and VG continuous tasks. Compared with the univariate analysis, e.g., amplitude of low-frequency fluctuation (ALFF) at each frequency band, including, wavelet-ALFF, the 1D-CNN model highly outperformed wavelet-ALFF, with more efficient decoding models [46% of 800 models showing area under the curve (AUC) > 0.61] and higher decoding accuracies (94% of the efficient models), especially on the high-frequency bands (>0.1 Hz). Moreover, our results also demonstrated the advantages of wavelet decompositions over the original fMRI series by showing higher decoding performance on all peak voxels. Overall, this study suggests a great potential of single-voxel analysis using 1D-CNN and wavelet transformation of fMRI series with continuous, naturalistic, steady-state task design or resting-state design. It opens new avenues to precise localization of abnormal brain activity and fMRI-guided precision brain stimulation therapy.

## Introduction

### Importance of Location Diagnosis by Functional Magnetic Resonance Imaging

Blood-oxygen-level-dependent (BOLD) fMRI has been widely used to investigate brain activity under cognitive tasks or resting state, which offers great opportunities to develop an objective assessment for the functional abnormalities of neurological disorders (Uddin et al., 2017; Lunkova et al., 2021). From a clinical perspective, brain imaging diagnosis includes at least three purposes, namely, qualitative diagnosis (i.e., the nature or what specific disease is, e.g., Parkinson's disease), location diagnosis (i.e., where precisely the abnormality is), and quantitative diagnosis (i.e., the extent of the abnormality). As one of the main applicable scenarios, fMRI technologies in combination with various analytic tools have been used in the field of location diagnosis, for instance, to localize the abnormal brain activity using functional task localizers (Kohls et al., 2014; Li et al., 2015, 2020) or to detect the difference of intrinsic or spontaneous brain activity at the voxel level using amplitude of low-frequency fluctuation (ALFF) (Pan et al., 2017; Gong et al., 2020).

### fMRI Analysis at Single Voxel Level for Location Diagnosis

For the typical task fMRI design, task stimuli were repeatedly presented to the participants in a manner of a blocked design or event-related design. To detect the activation pattern under a specific task condition, the general linear model (GLM) is used for modeling the block- or event-evoked activity of each single voxel. For resting-state fMRI (RS-fMRI), there are two categories of analytic methods, namely, one for functional integration, which measures the interaction or functional connectivity between brain regions, and the other for functional segregation, which measures the local activity, e.g., ALFF (Zang et al., 2007) of the time-series of each single voxel. Both the GLM and the ALFF analyze the fMRI data in a whole-brain voxel-wise manner, and hence, the results could be used for the subsequent coordinate- or voxel-based meta-analysis. For example, coordinate-based meta-analyses have found decreased task activation in the hippocampus (Li et al., 2015) and decreased RS-fMRI ALFF in the posterior cingulate cortex (Pan et al., 2017) in patients with mild cognitive impairment (MCI). Therefore, the “whole-brain voxel-wise” fMRI analytic methods could help precisely localizing the abnormal brain activity non-invasively.

### Limitations for Existing fMRI Design and Analysis

Both the repeated presented block and event-related fMRI designs are based on strong assumptions of GLM, e.g., additive and time invariant (Dale and Buckner, 1997). Moreover, many cognitive tasks do not meet the two strong hypotheses, e.g., naturalistic viewing or listening and continuously designed sustained attention task. Many researchers are using naturalistic experimental design (Hu et al., 2017; Mandelkow et al., 2017; Ren et al., 2018; Wen et al., 2018) or steady-state design (Chai et al., 2019) for the fMRI study. The primary difference between the newly developed naturalistic or steady-state fMRI designs and conventional block or even-related design is actually the data analysis. The analytic methods for naturalistic or steady-state fMRI design are very similar with those for RS-fMRI design, i.e., taking the whole time-series as a “continuous state” and then comparing between different states or different groups (e.g., patients vs. healthy controls). In this article, we would use “continuous-state” design to represent both naturalistic and steady-state design. As aforementioned, ALFF is one of the most popular methods for single-voxel analysis in RS-fMRI studies. Moreover, a few studies have applied ALFF to the continuous-state design (Dong et al., 2012; Zhang et al., 2021).

### Machine Learning at Single Voxel Level for fMRI

Currently, machine learning on multiple voxels, e.g., multivoxel pattern analysis (MVPA) (Haxby et al., 2001; Norman et al., 2006), or even on the voxels of the whole brain (Zhang et al., 2020a) has been widely used in fMRI studies. Most of these studies have reported more than 90% accuracy for either task fMRI decoding (Wang X. et al., 2020) or RS-fMRI classification of brain disorders (Hojjati et al., 2017; Zhang et al., 2017). Some articles reported the contribution of each voxel to the total diagnostic (Wang et al., 2007; Coutanche et al., 2011; Zhou et al., 2018). Moreover, yet this voxel-level contribution is only valid for a specific combination of multivoxels or even the whole region of interest, which is very different from the discriminative or diagnostic performance of a single voxel. In addition, MVPA utilizes the spatial coherence or spatial activation patterns to improve the prediction accuracy but ignores the temporal dynamics of each single voxel. In contrast, machine learning for single time-series has been used in the electrophysiological studies for classification of different sleep stages (Mousavi et al., 2019; Zhang et al., 2020b) and locating the most abnormal channel of epilepsy (Lu et al., 2018). However, to the best of our knowledge, no study has performed machine learning on the fMRI time-series of a single voxel. With the aid of machine learning, the performance can be highly improved, for instance, much better than using the conventional univariate analysis like performing *t*-test on activation values and hence, would be helpful for location diagnosis, i.e., a better precise localization of the abnormal activity.

### The Motivation of This Study

This study used convolutional neural network (CNN) at the single-voxel level to differentiate two “continuous-state” task design (sometimes named naturalistic task design) fMRI sessions. First, the time-series of a single voxel was extracted from the center of brain regions that showed robust activations in the population, which could be taken as “ground truth.” Then, instead of applying convolutional operations directly on these time-series, we used wavelet transformations to project brain activity into multiple frequency bands, which provides a time-frequency decomposition of brain activity across a continuous range of scales. Furthermore, we trained a one-dimensional (1D)-CNN classifier on the transformed time-series at each scale and evaluated their predictability among different brain states. Compared with the simple *t*-test of ALFF and 1D-CNN on original fMRI signals, the proposed method demonstrated higher detection rates and higher classification accuracies on all listed regions and generalized well across multiple subjects. Our results also indicated that not only the conventional frequency band of 0.01–0.08 Hz but also the higher frequency bands (>0.1 Hz) captured discriminative features of different brain states.

## Materials and Methods

### Participants

A total of 42 healthy participants (age: 24 ± 5.01 years, range 19–48; 22 female; all right-handed) were recruited in the study. They had no history of head trauma, substance abuse, or neuropsychiatric disorder. The study was approved by the Ethics Committee of the Center for Cognition and Brain Disorders at Hangzhou Normal University, and written informed consent was signed by all subjects before the experiment.

### Image Acquisition

The MRI data were acquired on a GE 3T scanner (MR-750, GE Systems, Milwaukee, WI) at the Center for Cognition and Brain Disorders of Hangzhou Normal University. The fMRI scanning sessions included an 8 min block-design task fMRI session and two 8 min continuous-state design task fMRI sessions with these parameters: slice number = 43, repetition time (TR) = 2,000 ms, TE = 30 ms, matrix = 64 × 64, voxel size = 3.44 × 3.44 × 3.2 mm, flip angle (FA) = 90°, field of view (FOV) = 220 × 220 mm.

### Finger Tapping Task: Blocked Design

**Figure 1:**
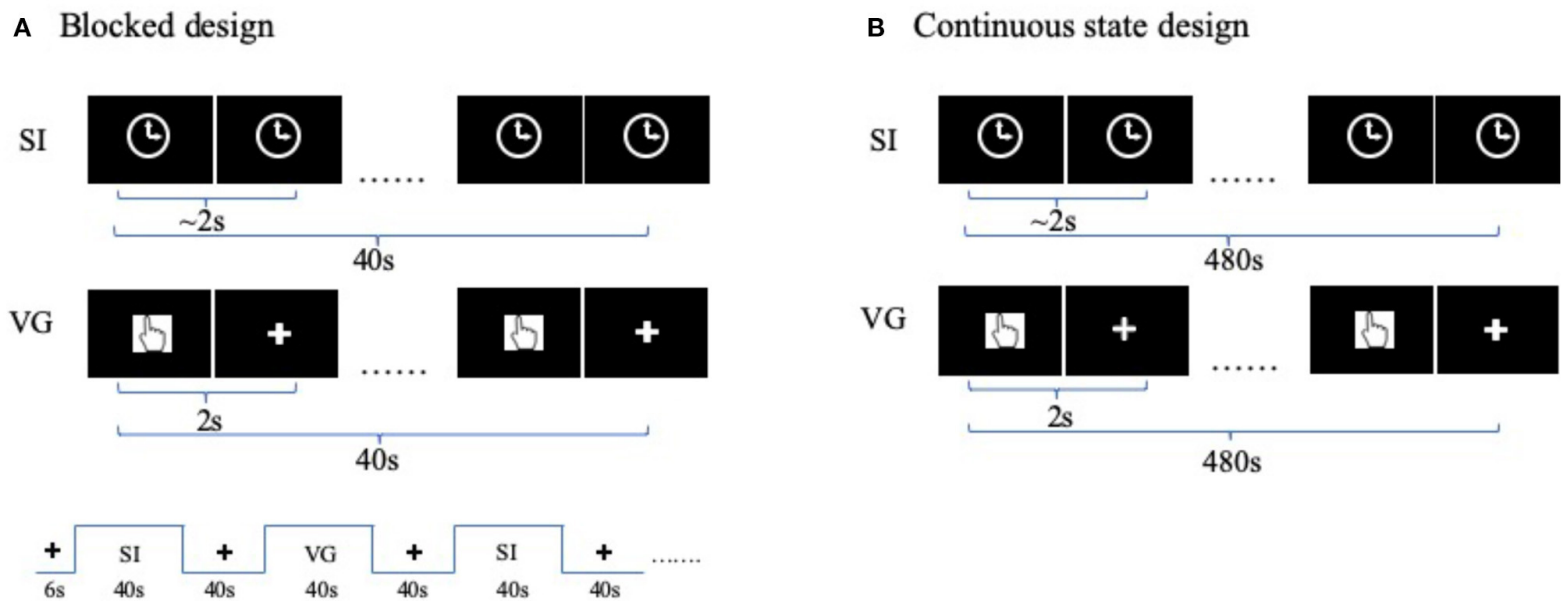
Task functional magnetic resonance imaging (fMRI) designs. **(A)** fMRI-blocked design for finger movement task. Each functional run includes three visual-guided (VG) task blocks, and three self-initiated (SI) task blocks, plus six rest blocks. In VG block, a visual cue of “finger” image was shown on the screen for every 2 s, indicating the time for the participant to press the button. In SI block, a clock sign was shown on the screen during the entire block. **(B)** fMRI continuous-state design. Participants performed each of the two tasks (i.e., SI and VG) for the entire functional run, each lasting for 8 min. The order of tasks is balanced between participants.

The 8 min blocked design finger tapping task contained visual-guided (VG) blocks (40 s × 3), self-initiated blocks (40 s × 3), and rest blocks (40 s × 6) (Wang J. et al., 2020). The VG task presented a visual cue of “finger” image for every 2 s followed by a fixation cross. The participant need to tap his/her finger once the finger image was presented. The SI task blocks presented a figure of clock during the entire task block. The participants need to perform finger tapping with a self-paced 2 s (Figure 1A).

### Finger Tapping Task: Continuous State Design

The continuous-state design is a continuous performing task. It is also named naturalistic design (Hu et al., 2017; Ren et al., 2018) or steady-state design (Chai et al., 2019) in light of the context. The finger-tapping task is the same as the blocked design, except that each VG and SI state lasted for 8 min in a separate run. Two orders of states are balanced between participants (Figure 1B).

### Preprocessing of fMRI Data

All functional MRI images were analyzed by using the toolbox named Data Processing Assistant for Resting-State fMRI (DPARSF) (Chao-Gan and Yu-Feng, 2010), which is based on Statistical Parametric Mapping (SPM) (http://www.fil.ion.ucl.ac.uk/spm). Specifically, we discarded the first several volumes (i.e., three volumes for the blocked design and five volumes for the continuous-state design, accounting for 6 and 10 s, respectively) in each functional run, due to signal stabilization. The preprocessing included the following: (1) slice timing; (2) realignment; (3) regressing out nuisance signals including 24 head motion parameters and signals of white matter and cerebrospinal fluid; (4) spatial normalization to the standard Montreal Neurological Institute (MNI) template (resampled into 3 × 3 × 3 mm); and (5) spatial smoothing with an isotropic Gaussian kernel of 6 mm full-width-at-half-maximum (FWHM) in block-design paradigm while with an isotropic Gaussian kernel of 4 mm in continuous-state design (smaller FWHM reduced the impacts from the neighbor voxels). None of the subjects was excluded due to excessive head motion based on the criteria of >2 mm displacement or an angular rotation of >2° in any direction.

### Task fMRI Activation

We used SPM12 for task activation analysis at both the subject (with high-pass filtering > 1/128 Hz) and group levels (using statistical analysis). Specifically, at the individual level, we applied the GLM to generate the activation maps under SI/VG conditions after regressing out head motion parameters (i.e., three parameters for translation and three for rotation). The contrast maps of SI minus VG condition (SI–VG) were then created for each subject and used to detect the significant differences in brain activations *via* one sample *t*-tests across all subjects [Gaussian random field (GRF) correction with voxel *p* < 0.001, cluster *p* < 0.05].

### Wavelet Transform of Continuous-State fMRI for Analysis

The SI and VG motor tasks have been widely used in task fMRI motor control studies, and their activation difference is well-validated in the literature (Hoffstaedter et al., 2013). Thus, we extracted the preprocessed fMRI time-series of the continuous state design in light of the peak voxels that have showed significant different activations in the two conditions in the blocked design. Instead of directly classifying brain states based on original fMRI time-series, we applied the continuous wavelets transforms (CWTs) to the time-series to obtain a time-frequency decomposition across a continuous range of scales. Wavelets have become an important tool in the fMRI analysis (Bullmore et al., 2004) not only for connectivity analysis of healthy populations (Guo et al., 2012) but also for detecting biomarkers of neurological diseases (Wang et al., 2013).

The CWT coefficient is defined as the convolution of the blood-oxygen-level-dependent (BOLD) signal *x(t)* with the scaled and translated version of the mother wavelet ψ*(t)* as shown in equation (1):


(1) $$\begin{array}{l} CWT\left(a,b\right)={\left|a\right|}^{-1/2}\int x\left(t\right)\psi \left(\frac{t-b}{a}\right)dt \end{array}$$

where *a* denotes the wavelet scale, *b* denotes the time shift position, and ψ(*t*) represents the mother wavelet, for instance, Wavelet Daubechies 2 (db2) (Daubechies, 1990; Übeyli, 2009). Previous studies have shown that the db2 has higher superiority than other bases for BOLD signal (Zhang et al., 2016; Luo et al., 2020).

**Figure 2:**
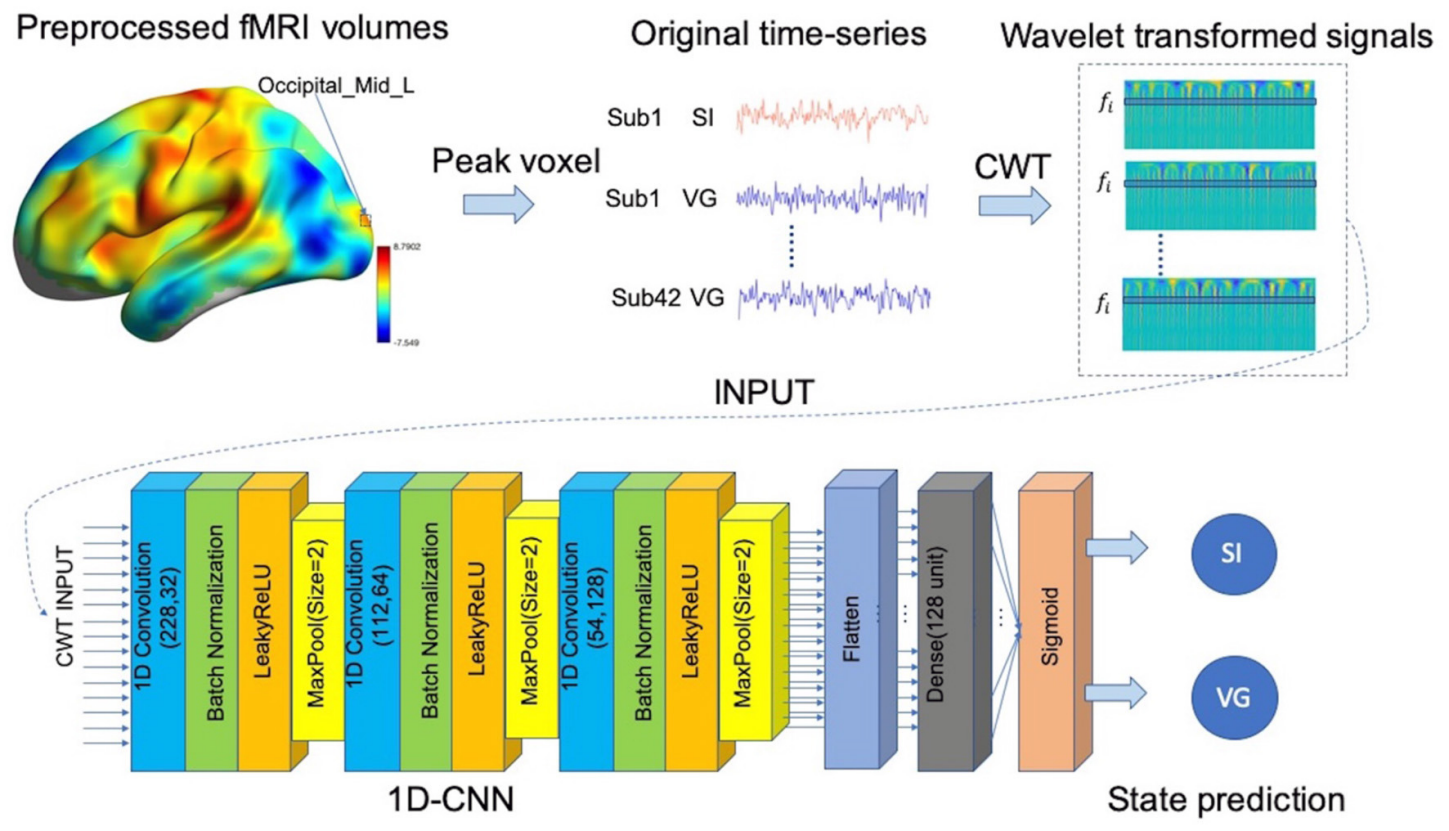
Pipeline of hybrid 1D convolutional neural network (1D-CNN) prediction model. We first extracted the blood-oxygen-level-dependent (BOLD) signal from a specified target voxel (e.g., Occiptial_Mid_L: left middle occipital gyrus) for each subject under each condition (i.e., SI or VG). We then applied continuous wavelet transforms (CWTs) to the time-series to generate time-frequency decomposition across continuous scales. The transformed time-series of each frequency band were then imported to the 1D-CNN model to predict the continuous state.

As shown in Figure 2, for each predefined target voxel, the BOLD time-series was first extracted from preprocessed fMRI datasets and then transformed into the time-frequency domain by using db2 *via* CWT function in MATLAB2018. As a result, we obtained 32 copies of wavelet coefficients at different frequency bands, within the range of 0.003–0.313 Hz with an interval of 0.01 Hz, in total of 84 × 32 × 230 values, where 84 is the sample size (i.e., 42 subjects and two states for each subject), 32 is the number of frequency bands, and 230 is the time points (coefficients). These wavelet coefficients were then treated as features to classify brain states (i.e., VG and SI), with a separate 1D-CNN model for each frequency bands (see the “1D convolutional neural networks (1D-CNN) on fMRI signals” section). As a baseline approach, we also calculated the amplitude of low fluctuation based on the wavelet coefficients, including wavelet-ALFF (Luo et al., 2020), and evaluated their predictability of VG and SI states.

### 1D Convolutional Neural Networks on fMRI Signals

After converting BOLD signals to scalogram images using CWT, i.e., maps of wavelet coefficients at different frequency bands, based on the task states, the data were split into 75 and 25% for training and testing, respectively. Each frequency band in scalogram was then imported into the 1D-CNN model consisting of three convolutional blocks (i.e., 32, 64, and 128 filters) and followed by a flatten layer and a fully connected layer (128 units). Each convolutional block consists of one convolutional layer (kernel size = 3), one non-linearity layer (LeakyReLU: alpha = 0.3), and one pooling layer. Due to the small sample size for model training, considerable strategies were applied to overcome model overfitting, including batch normalization, dropout at dense layer, and sparsity regularization. We used Adam as the optimizer with the initial learning rate as 0.00005. Additional l1 regularization of 0.00001 on weights was used to control model overfitting and the noise effect of fMRI signals. The dropout rate of 0.5 was additionally applied to the dense layer.

We chose the “binary_crossentropy” as the loss function. Other hyperparameters are set as follows: batch size = 2; optimize function: Adam; initial learning rate: 0.001; kernel_initializer: “he_uniform” and bias_initializer: “zeros” for the Convolutioan1D layer; kernel_initializaer: “lecun_normal” for the dense layer. We used the training set to train model parameters and saved the best model with the highest prediction score on the test set after 200 training epochs. The whole process was repeated for 10 times, considering the effects of different random initializers of model parameters. The average classification score over 10 repetitions was reported as the final decoding performance. For comparison, the same train-test split of scalogram images was used to calculate the wavelet-ALFF.

### Performance Evaluation

**Figure 3:**
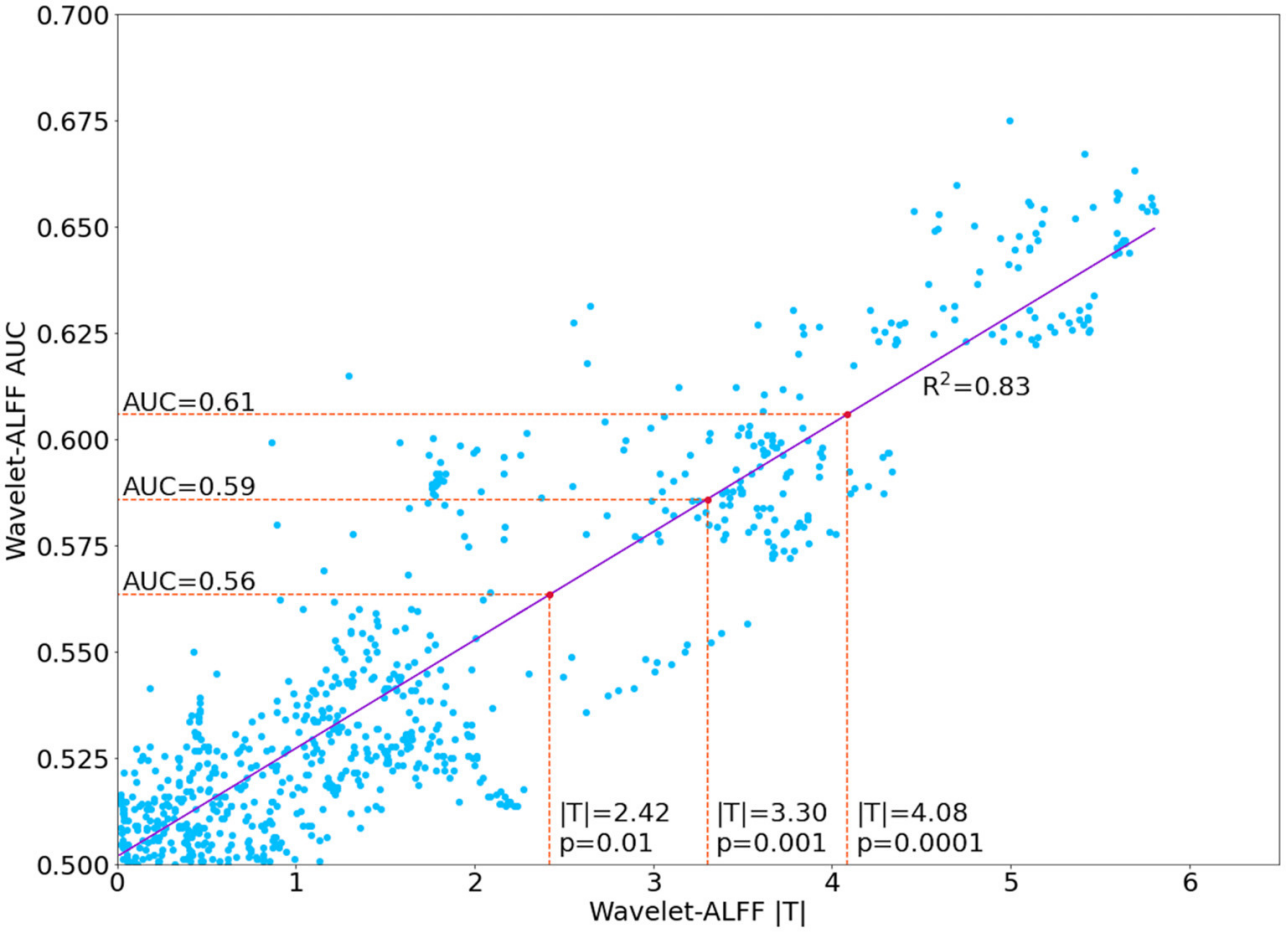
Linear correlation of between the area under the curve (AUC) of wavelet amplitude of low frequency fluctuation (wavelet-ALFF) and the |T| value of the paired *t*-test between two continuous task states (i.e., SI vs. VG).

The decoding performance of SI and VG states was evaluated by using area under the curve (AUC), which provides an aggregate measure of performance across all possible classification thresholds. The AUC represents the probability that the model ranks a random positive (i.e., SI state) example more highly than a random negative (i.e., VG state) example. In this study, we used the difference in wavelet-ALFF values as the baseline and compared it with the classification performance of 1D-CNN. In addition, for each frequency band and each peak voxel, we applied paired *t*-tests on the wavelet-ALFF scores and found an association between the statistical tests (i.e., T-score in paired *t*-test) and classification accuracies (i.e., AUC). Specifically, a linear regression model was fitted between the absolute |T| values and the AUCs of state classification (model fitness: *R*^2^ = 0.83). As a result, each AUC score can be transferred into a T-score *via* the regression model, for instance, |T| value of 4.08 (*p* < 0.0001) was corresponding to an AUC of 0.61 (as shown in Figure 3). Thus, all decoding models with AUC > 0.61 were considered as efficient predictions of brain states in both wavelet-ALFF and 1D-CNN approaches.

## Results

### Difference Between SI and VG Tasks

**Figure 4:**
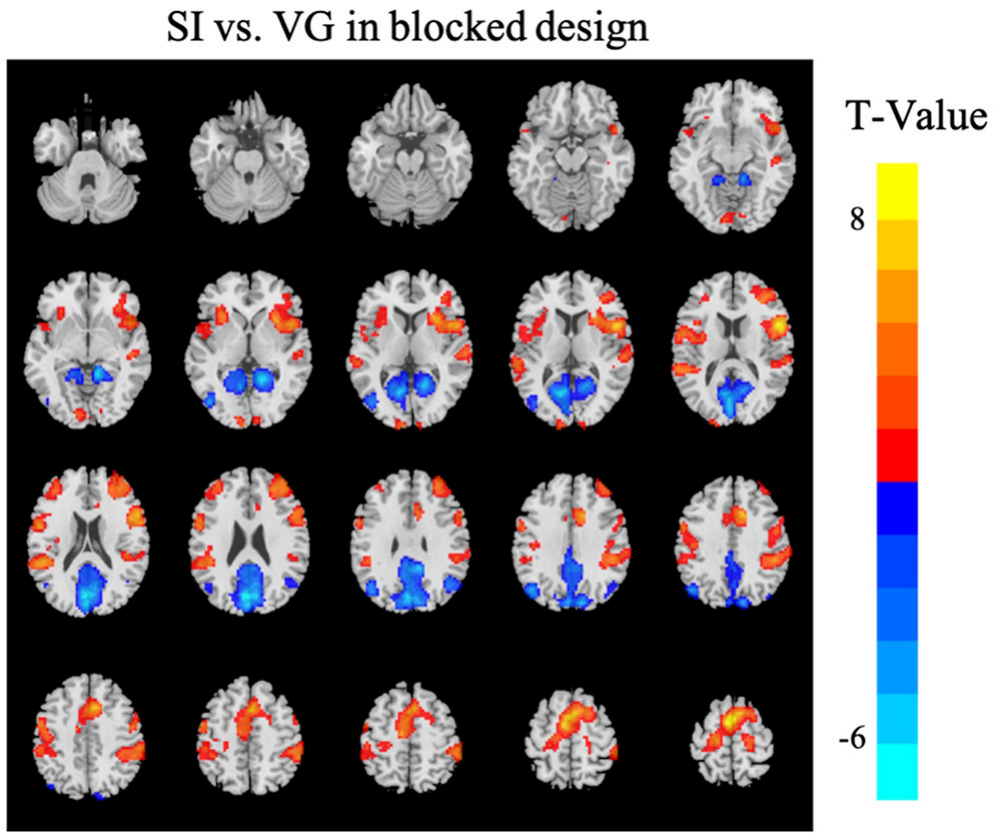
Differences in brain activations between SI and VG tasks in the blocked design [Gaussian random field (GRF) corrected with voxel *p* < 0.001 and cluster *p* < 0.05]. Warm colors (red) indicate higher brain activation or activity in the SI condition than VG; cool colors (blue) indicate higher brain activation or activity in the VG condition than SI. The Z-coordinates were from −30 to +65 with a step of 5 mm. Left in the figure is the left in the brain.

The task activation analysis in the blocked design showed higher activation during SI task in the bilateral primary sensorimotor cortices, supplementary motor cortex, dorsal anterior cingulate cortex, and anterior insular, while higher activation during VG task in the precuneus and visual cortex (corrected for multiple comparison using GRF with voxel *p* < 0.001, cluster *p* < 0.05; Figure 4). A full list of brain regions along with the peak coordinates can be found in Table 1.

**Table 1:** Coordinates of brain activation of self-initiated (SI) vs. visual-guided (GV) motor tasks in the blocked design.

**ID**	**Brain regions**	**Number of voxels**	***T*-value**	**MNI coordinates**		
				***X***	***Y***	***Z***
**SI < VG**						
1	Cuneus_L	223	−7.55	3	78	24
2	Lingual_R	126	7.04	18	51	3
3	Calcarine_L	244	6.89	12	66	9
4	Cuneus_R	188	6.87	6	78	27
5	Calcarine_R	127	6.62	18	54	6
6	Lingual_L	117	6.33	9	63	6
7	Precuneus_R	123	5.82	6	66	24
8	Cingulum_Post_L	72	5.65	0	45	30
9	Precuneus_L	96	5.61	6	54	30
**SI > VG**						
10	Temporal_Sup_R	72	5.82	60	27	12
11	Parietal_Inf_R	78	5.95	57	36	48
12	Frontal_Sup_R	65	6.15	18	12	63
13	Occipital_Mid_L	85	6.19	9	105	3
14	Postcentral_L	111	6.19	57	0	18
15	Frontal_Mid_R	237	6.21	30	45	24
16	SupraMarginal_R	151	6.33	48	36	39
17	Precentral_L	128	6.75	18	21	69
18	Insula_R	130	6.87	30	18	6
19	Cingulum_Mid_R	126	7.33	9	15	45
20	Temporal_Sup_L	115	7.57	51	39	18
21	Precentral_R	92	7.68	57	9	18
22	Supp_Motor_Area_R	240	7.96	3	3	66
23	Rolandic_Oper_R	93	8.14	54	6	15
24	Frontal_Inf_Oper_R	166	8.49	51	9	12
25	Supp_Motor_Area_L	307	8.79	3	0	66

*The names of brain regions are from the automated anatomical labeling (AAL) template (Tzourio-Mazoyer et al., 2002). L, left; R, right; Post, posterior; Sup, superior; Inf, inferior; Mid, middle; Supp, supplementary; Oper, operculum. Brain region, coordinates, volume, and peak t value were reported by SPM12 (http://www.fil.ion.ucl.ac.uk/spm)*.

### Classification Performance of 1D-CNN SI and VG of Continuous States

We trained a series of 1D-CNN models to decode continuous states for each frequency band and each peak voxel. Each model was trained on the same train-test split for ten times by taking into account the impact of random initializations and reported the average decoding accuracy as the final decoding performance.

**Figure 5:**
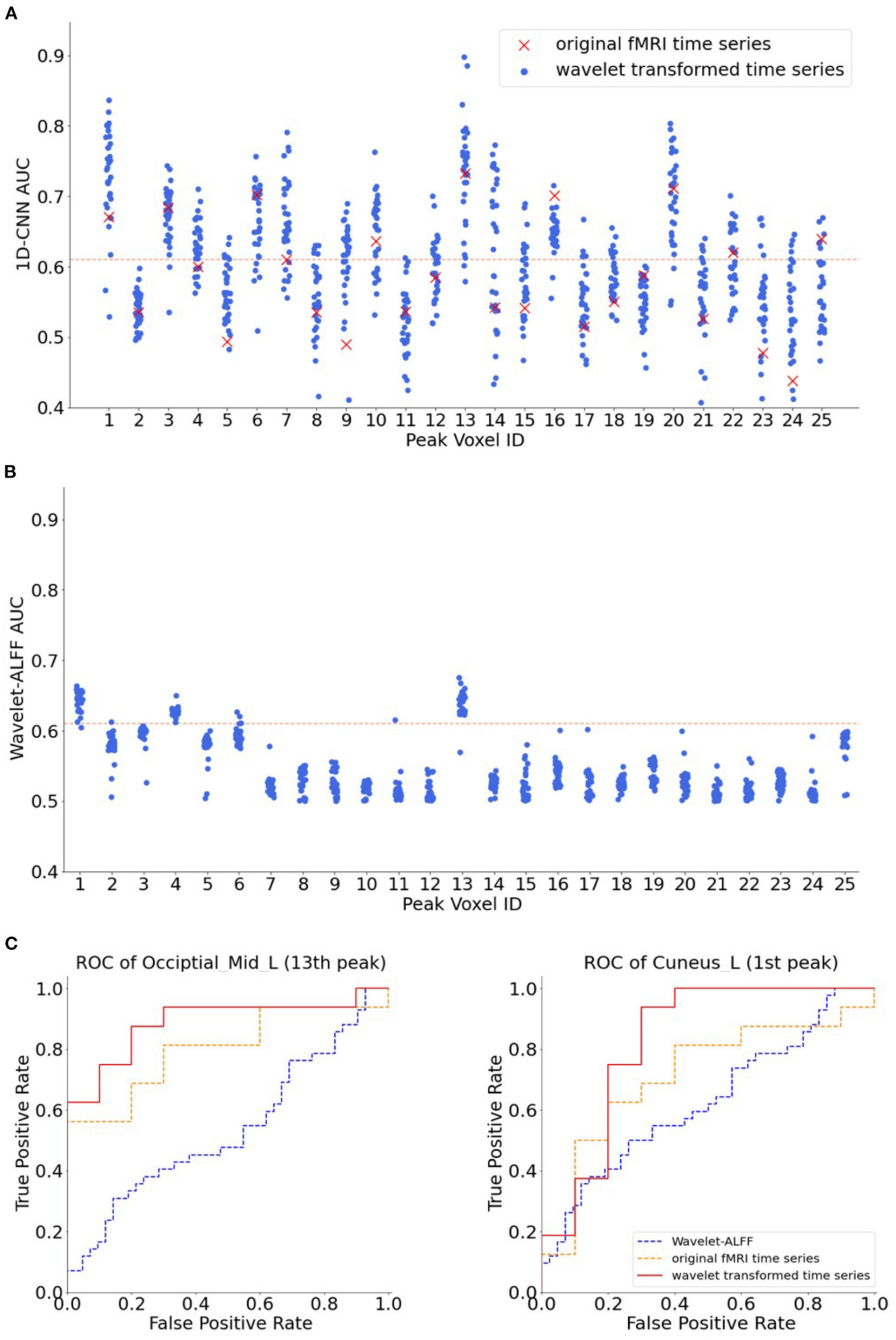
The AUC of 25 peak voxels. The names of each peak voxel were listed in Table 1. **(A)** Decoding performance of 1D-CNN models on the transformed time-series across 32 frequency bands as well as on the original functional magnetic resonance imaging (fMRI) time-series. **(B)** Decoding performance of wavelet-ALFF across 32 frequency bands. The read dashed lines indicate the threshold of AUC > 0.61 (corresponding to paired |T| > 4.08 and *p* < 0.0001). The red crosses in A indicate the AUCs of original fMRI time-series, with a mean rank of 20.4 across the 25 voxels, i.e., below the average performance of 1D-CNN. Both the 1D-CNN and wavelet-ALFF models showed the highest decoding performance in the 13th peak (Occipital_Mid_L in Table 1) and the second best performance in the 1st peak (Cuneus_L in Table 1). **(C)** Receiver operating characteristic (ROC) of 1D-CNN on two exemplar peak voxels, i.e., the 13th peak (Occipital_Mid_L, in the left panel) and the 1st peak (Cuneus_L, in the right panel). Red lines in the plots indicate the ROC of 1D-CNN on wavelet-transformed time-series at a specific frequency. Note that different frequencies were chosen for the two peak voxels according to their best performance, namely, 0.233 and 0.083 Hz, respectively. Blue lines and orange lines indicate the ROC of wavelet-ALFF and original fMRI time-series, respectively.

To evaluate the benefits of using the wavelet scalogram of BOLD signals, we tested different temporal features in 1D-CNNs, including wavelet-transformed time-series at each frequency band, as well as the original fMRI time-series, in total of 33 × 25 = 825 decoding models. We found that the 1D-CNN models on wavelet coefficients highly outperformed the original fMRI time-series (Figure 5A). The average rank of decoding performance using the original time-series was 20.4 out of 33 models among all 25 peak voxels, even lower than the mean accuracy of CWT across all frequency bands (16 out of 33 models).

When fixing the features as the scalogram maps, we found that the 1D-CNN models highly outperformed the wavelet-ALFF among all frequency bands and peak voxels (Figure 5). First, the two approaches revealed some common regions in the visual cortex that showed high predictive power on continuous task states, for instance, areas in the occipital lobe (13th peak: Occipital_Mid_L and 1st peak: Cuneus_L). More importantly, the 1D-CNN models uncovered a variety of new regions in the frontal (12th peak: Frontal_Sup_R) and temporal lobes (20th peak: Temporal_Sup_L), as well as motor areas (22nd peak: Supp_Motor_Area_R), which showed efficient predictions of brain states only in the 1D-CNN models but not in wavelet-ALFF.

**Figure 6:**
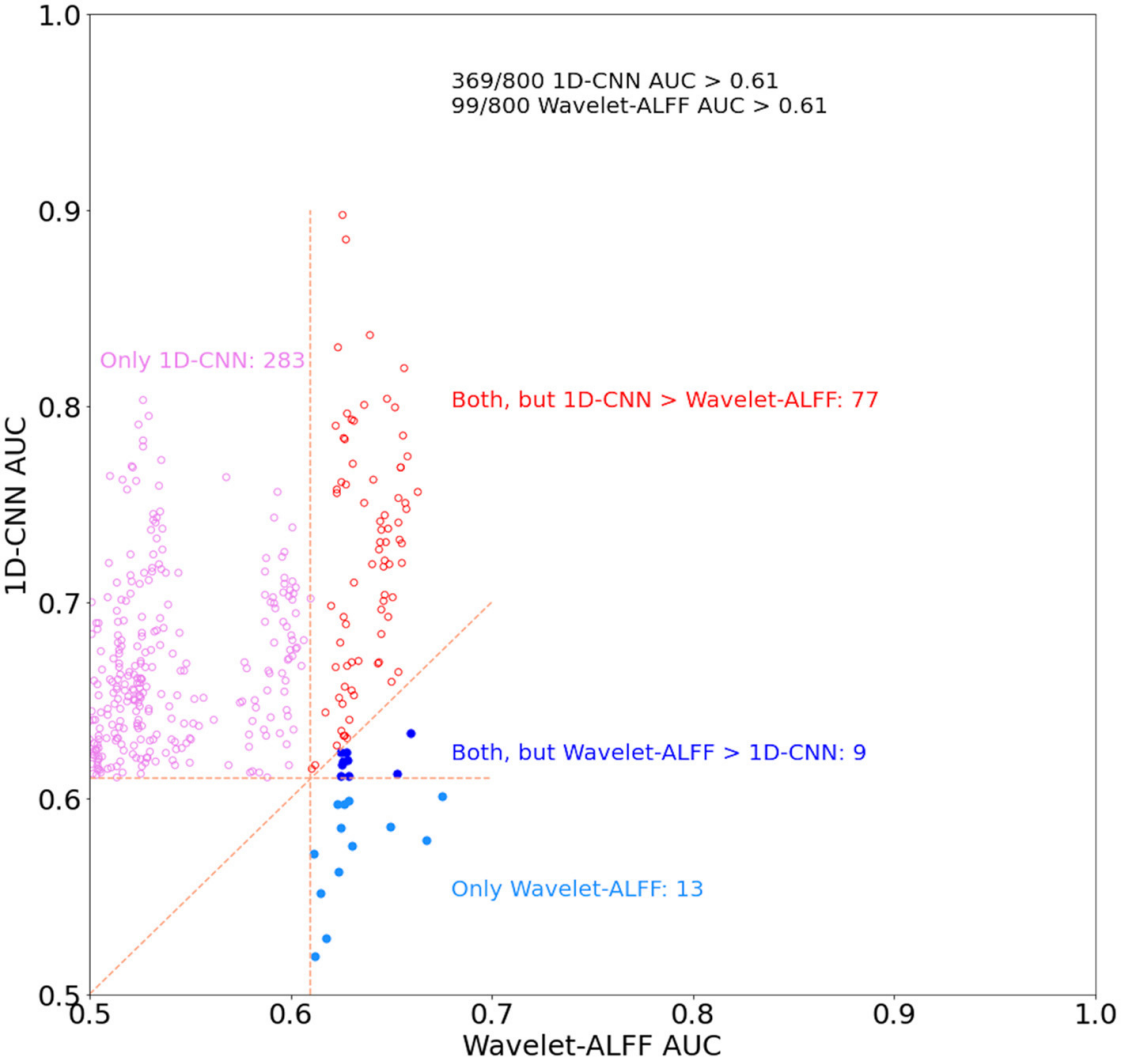
The relationship of decoding performance in the 1D-CNN and wavelet-ALFF models. We used area under the curve (AUC) to evaluate the decoding of continuous task states for both methods and only the models with AUC > 0.61, corresponding to |T| > 4.08 (*p* < 0.0001) in the paired *t*-test on wavelet-ALFF decoding models. There were 800 pairs of decoding models in total across 32 frequency bands and 25 peak voxels. 1D-CNN models showed more efficient predictions of task states than wavelet-ALFF (369 vs. 99 models, respectively) as well as higher decoding accuracies (94% of efficient models).

Second, the quantitative comparison of the two approaches revealed that the 1D-CNN models showed higher prediction accuracies than wavelet-ALFF (Figure 6). Notably, to control false-positive rates, in this analysis, we only considered the decoding models that efficiently predict task states, i.e., with AUC > 0.61 (corresponding to |T| > 4.08 and *p* < 0.0001 in the paired *t*-test results on wavelet-ALFF, as shown in Figure 3). There were 800 pairs of wavelet-ALFF and 1D-CNN models in total (25 peak voxels × 32 frequency bands). As shown in Figure 6, 46% (369 out of 800 models) of 1D-CNN models showed efficient predictions of task states (AUC > 0.61, maximum value = 0.9). In contrast, only 12% (99 out of 800 models) of wavelet-ALFF models showed efficient predictions (AUC > 0.61, maximum value = 0.67). The overlapping area only consists of 86 models, i.e., AUCs > 0.61 for both 1D-CNN and wavelet-ALFF models. Among which, more than 90% cases (77 out of 86 models) indicated a higher prediction in 1D-CNN than wavelet-ALFF. On the other hand, there were, in total, 382 models that showed efficient predictions in either 1D-CNN or wavelet-ALFF, 94% of which showed higher AUCs in 1D-CNN and 6% was higher in wavelet-ALFF.

**Figure 7:**
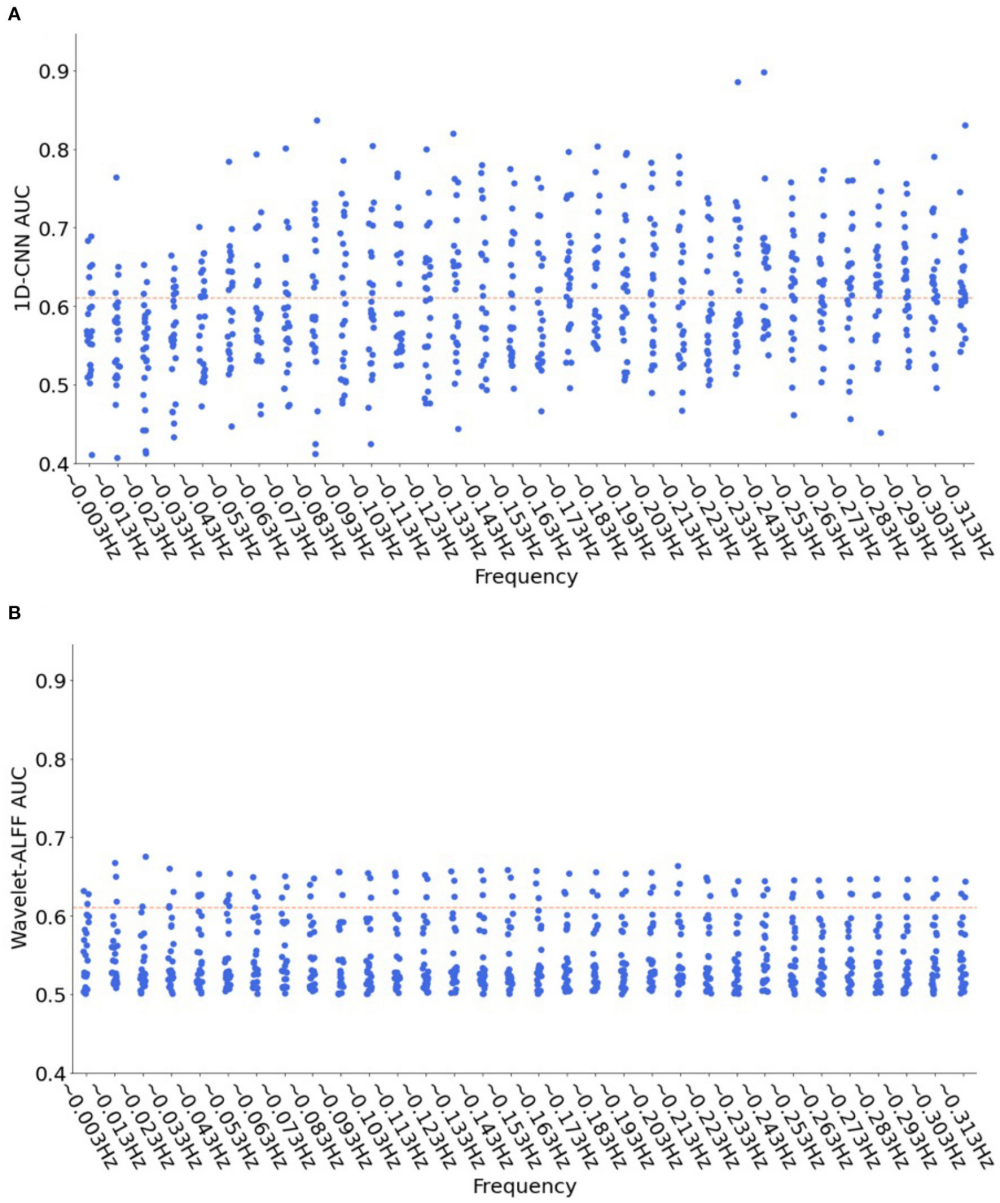
Distribution of decoding performance across 32 frequency bands for both 1D-CNN **(A)** and wavelet-ALFF **(B)**. Each dot in the figure represents the AUC results of 1D-CNN **(A)** or wavelet-ALFF **(B)** for each peak voxel at each frequency band, in total of 25 (peak voxels) × 32 (frequency bands) = 800 dots. The read dashed lines indicate the efficient predictions with a threshold of AUC > 0.61 (corresponding to |T| > 4.08 and *p* < 0.0001). The 1D-CNN showed better predictions on high frequency bands (>0.1 Hz), while wavelet-ALFF showed more uniform distribution with a preference on low frequency bands (<0.1 Hz).

Moreover, to show more frequency-specific characteristics, we plotted the decoding performance of the 1D-CNN models and wavelet-ALFF across 32 frequency bands (Figure 7). The results indicated that the efficient predictions (AUC > 0.61) of 1D-CNN were mostly located in the high frequency bands (>0.1 Hz). In contrast, the prediction of wavelet-ALFF was more uniformly distributed across frequency bands, with a trend of better predictions in the lower frequency bands (<0.1 Hz).

## Discussion

In this study, we aimed to decode continuous task states by using the temporal dynamics of a single voxel from fMRI recordings. Taking the activation maps from the blocked design task as reference, 25 peak voxels were identified, and their fMRI time-series were transformed into the temporal-frequency domain using wavelet. We applied a hybrid 1D-CNN on the transformed time-series at each frequency band, as well as the original fMRI time-series, to decode SI and VG continuous task states. Our results showed that the hybrid 1D-CNN model successfully decoded continuous task states by only using on fMRI time-series from a single voxel, with the highest AUC reaching almost 90% on the middle occipital lobe. The proposed model highly outperformed the conventional univariate analysis, e.g., wavelet-ALFF, not only showing more efficient predictions across all selected peak voxels and frequency bands but also achieving higher decoding performance in the same settings. Moreover, our model achieved better decoding by using the wavelet transformed time-series than original fMRI time-series on all peak voxels.

This study focused on machine learning on fMRI time-series at a single voxel. In previous fMRI studies, the majority of machine learning approaches aimed to differentiate two groups of people (e.g., patients and healthy controls) or two conditions (e.g., two task condition or two resting-state conditions) by using imaging data from multiple voxels or even the whole brain. This type of analysis is something like “qualitative diagnosis” in clinical radiology, i.e., what kind of disease it is. Another even more important clinical aspect is location diagnosis, i.e., where precisely the abnormality is. The location diagnosis not only helps the qualitative diagnosis but also provides the guidance to the precise brain stimulation therapy, e.g., deep brain stimulation, focused ultrasound stimulation, and transcranial magnetic stimulation. fMRI brain imaging holds advantages of non-invasiveness, fairly high spatial and temporal resolution, and easy access; therefore, it is a promising tool for precise localization of abnormal brain activity. To the best of our knowledge, this is the first study of machine learning on the single-voxel analysis of task fMRI data. We applied 1D-CNN on the wavelet-transformed time-series at a specific frequency band to predict the continuous task states of the human brain and found much better performance than the univariate analysis, e.g., wavelet-ALFF, not only in the visual cortex including the middle occipital lobe and cuneus but also in the SMA, prefrontal and temporal regions that have been well-validated in self-initiated movements (Zimnik et al., 2019). We believed that machine learning on single-voxel analysis could be applied to brain disorders and hence to help precise localization of the abnormal brain activity.

In addition to the comparison with univariate analysis, i.e., wavelet-ALFF, we also compared the performance of 1D-CNN on the original fMRI time-series with the wavelet-transformed time-series of 32 frequency bands. We found that the mean rank of 1D-CNN decoding performance on the original fMRI time-series was 20.4 across the 25 peak voxels, below the average performance on the wavelet transformed time-series (mean range is 16). Moreover, 1D-CNN on the transformed time-series outperformed the original time-series on all selected peak voxels. CNN has been widely applied in the time-series analysis, ranging from forecasting to classification of brain activity (Acharya et al., 2018; Kam et al., 2019; Mousavi et al., 2019). In most cases, the researchers relied on the neural networks and backpropagation of errors to learn efficient features from the time-series. These models have also showed acceptable performance on the task of interest, 0.5–0.75 in our case. However, in this study, we demonstrated that the performance of deep neural networks can be further improved if our domain knowledge is also applied. A series of fMRI studies revealed that the brain activity at different frequency bands showed various contributions to neural interactions during task execution (Sasai et al., 2021). For example, low-frequency (<0.1 Hz) fluctuation is highly correlated among sensorimotor cortices (Biswal et al., 1995), very-low-frequency band <0.01 Hz (Slow-6) activity in bilateral basal ganglia is related to the performance of motor feedback (Zhang et al., 2015), and relative high-frequency band (>0.15 Hz) detects task-specific functional connectivity patterns in the prefrontal cortex during the visual task and the heart task (Sasai et al., 2021). In this study, we used wavelet transform to extract the frequency-specific characteristics in the fMRI time-series and achieved much better decoding performance of task states than the original time-series, especially in the high-frequency bands (>0.1 Hz). One possible explanation is that fMRI activity at a single voxel is usually contaminated by noise from various physiological processes. The wavelet transformation decomposed the fMRI signals into the time-frequency domain at continuous scales, which not only partially improved the ratio of signal-to-noise ratio (SNR) of the transformed signals but also captured more task-related dynamics by extracting time-series at a specific frequency band and therefore, boosted the decoding performance of neural networks.

It is worth mentioning that a continuous-state paradigm was used in this task fMRI study. Conventional task fMRI activation studies usually use blocked design or event-design, which requires repeated blocks or random events. The corresponding data analytic approach requires strong assumptions of GLM, e.g., additive and time invariant. In the past years, an increasing body of fMRI studies has started to explore cognitive processes under naturalistic experimental design (Hu et al., 2017; Mandelkow et al., 2017; Ren et al., 2018; Wen et al., 2018) or steady-state design (Chai et al., 2019) in light of the context. We used blocked design for task fMRI activation analysis. Then, we used continuous-state design of task fMRI for the 1D-CNN analysis. We used the term of “continuous state” because the task is not that “naturalistic” or “steady.” Anyway, all “naturalistic” design, “steady-state” design, and “continuous-state” design are very similar with “resting-state” design: (1) They are a relatively long “continuous state” (a few minutes on longer) as compared with the alternative blocks or events; (2) they share similar analytic methods that are widely used in RS-fMRI studies including functional connectivity or network analysis or ALFF or ReHo; and (3) they are very different from the signal contrast evoked by task blocks or events in the conventional task fMRI design. This study used 1D-CNN for the classification of two task states at each single voxel, hence, help the precise localization of brain abnormalities in clinic applications. This method could be also used in naturalistic, steady-state, and RS-fMRI design.

A few limitations should be addressed. First, there were only 42 participants in this study, each going through two continuous task states. The generalizability of this method needs to be tested in a larger sample size of participants. Second, due to the limitation of computation capacity, only 25 peak voxels were selected. Future studies could perform a voxel-wise 1D-CNN in the whole brain. Third, this study was based on continuous task state fMRI data. However, RS-fMRI data are more useful for clinical studies. Whether this idea could be applied to RS-fMRI data is still unknown. Fourth, the 1D-CNN performance in this study is not high. More comprehensive classification methods should be developed for single-voxel analysis of fMRI time-series and hence help precise localization of abnormal brain activity.

## Conclusion

The 1D-CNN classification on the wavelet-transformed time-series successfully differentiated the two continuous states of fMRI tasks, SI vs. VG tasks, and highly outperformed the conventional univariate wavelet-ALFF, as well as the 1D-CNN on original fMRI time-series. With a combination of 1D-CNN and wavelet transformation, our results demonstrate the feasibility of the single-voxel analysis of fMRI data for decoding cognitive states. This study shows great potentials for precise localization of abnormal brain activity and fMRI-guided precision brain stimulation therapy.

## Data Availability Statement

The raw data supporting the conclusions of this article will be made available by the authors, without undue reservation.

## Ethics Statement

The studies involving human participants were reviewed and approved by Ethics Committee of the Center for Cognition and Brain Disorders (CCBD) at Hangzhou Normal University. The patients/participants provided their written informed consent to participate in this study.

## Author Contributions

All authors listed have made a substantial, direct, and intellectual contribution to the work and approved it for publication.

## Funding

This study was supported by NSFC (82071537 and 81520108016), Key Realm R&D Program of Guangdong Province (2019B030335001), Key Medical Discipline of Hangzhou, and the Cultivation Project of the Province-leveled Preponderant Characteristic Discipline of Hangzhou Normal University (18JYXK046 and 20JYXK004). YZ was partially supported by the Major Scientific Project of Zhejiang Lab (Nos. 2020ND8AD01 and 2021ND0PI01).

## Conflict of Interest

The authors declare that the research was conducted in the absence of any commercial or financial relationships that could be construed as a potential conflict of interest.

## Publisher's Note

All claims expressed in this article are solely those of the authors and do not necessarily represent those of their affiliated organizations, or those of the publisher, the editors and the reviewers. Any product that may be evaluated in this article, or claim that may be made by its manufacturer, is not guaranteed or endorsed by the publisher.

## References

[B1] Acharya U. R. Oh S. L. Hagiwara Y. Tan J. H. Adeli H. (2018). Deep convolutional neural network for the automated detection and diagnosis of seizure using EEG signals. Comput. Biol. Med. 100, 270–278. 10.1016/j.compbiomed.2017.09.017 28974302

[B2] Biswal B. Yetkin F. Z. Haughton V. M. Hyde J. S. (1995). F1000Prime recommendation of: functional connectivity in the motor cortex of resting human brain using echo-planar MRI. Magn Reson Med. 10.1002/mrm.1910340409 8524021

[B3] Bullmore E. Fadili J. Maxim V. Sendur L. Whitcher B. Suckling J.. (2004). Wavelets and functional magnetic resonance imaging of the human brain. Neuroimage 23, 234–249. 10.1016/j.neuroimage.2004.07.012 15501094

[B4] Chai Y. Handwerker D. A. Marrett S. Gonzalez-Castillo J. Merriam E. P. Hall A.. (2019). Visual temporal frequency preference shows a distinct cortical architecture using fMRI. Neuroimage 197, 13–23. 10.1016/j.neuroimage.2019.04.048 31015027 PMC6591056

[B5] Chao-Gan Y. Yu-Feng Z. (2010). DPARSF: A MATLAB toolbox for “pipeline” data analysis of resting-state fMRI. Front. Syst. Neurosci. 4, 1–7. 10.3389/fnsys.2010.00013 20577591 PMC2889691

[B6] Coutanche M. N. Thompson-Schill S. L. Schultz R. T. (2011). Multi-voxel pattern analysis of fMRI data predicts clinical symptom severity. Neuroimage 57, 113–123. 10.1016/j.neuroimage.2011.04.016 21513803 PMC3105443

[B7] Dale A. M. Buckner R. L. (1997). Selective averaging of rapidly presented individual trials using fMRI. Hum. Brain Mapp. 5, 329–340. 10.1002/(SICI)1097-0193(1997)5:5<329::AID-HBM1>3.0.CO;2-5 20408237

[B8] Daubechies I. (1990). The wavelet transform, time-frequency localization and signal analysis. IEEE Transact. Inf. Theory 36, 961–1005. 10.1109/18.5719915843729

[B9] Dong Z. Y. Liu D. Q. Wang J. Qing Z. Zang Z. X. Yan C. G.. (2012). Low-frequency fluctuation in continuous real-time feedback of finger force: a new paradigm for sustained attention. Neurosci. Bull. 28, 456–467. 10.1007/s12264-012-1254-2 22833043 PMC5561895

[B10] Gong J. Wang J. Qiu S. Chen P. Luo Z. Wang J.. (2020). Common and distinct patterns of intrinsic brain activity alterations in major depression and bipolar disorder: voxel-based meta-analysis. Transl. Psychiatry 10. 353–366 10.1038/s41398-020-01036-5 33077728 PMC7573621

[B11] Guo C. C. Kurth F. Zhou J. Mayer E. A. Eickhoff S. B. Kramer J. H.. (2012). One-year test-retest reliability of intrinsic connectivity network fMRI in older adults. Neuroimage 61, 1471–1483. 10.1016/j.neuroimage.2012.03.027 22446491 PMC4226138

[B12] Haxby J. V. Gobbini M. I. Furey M. L. Ishai A. Schouten J. L. Pietrini P. (2001). Haxby2001Science. Science 293, 2425–2430. 10.1126/science.1063736 11577229

[B13] Hoffstaedter F. Grefkes C. Zilles K. Eickhoff S. B. (2013). The “what” and “when” of self-initiated movements. Cerebr. Cortex 23, 520–530. 10.1093/cercor/bhr391 22414772 PMC3593700

[B14] Hojjati S. H. Ebrahimzadeh A. Khazaee A. Babajani-Feremi A. (2017). Predicting conversion from MCI to AD using resting-state fMRI, graph theoretical approach and SVM. J. Neurosci. Methods 282, 69–80. 10.1016/j.jneumeth.2017.03.006 28286064

[B15] Hu X. Guo L. Han J. Liu T. (2017). Decoding power-spectral profiles from FMRI brain activities during naturalistic auditory experience. Brain Imaging Behav. 11, 253–263. 10.1007/s11682-016-9515-8 26860834

[B16] Kam T.-E. Wen X. Jin B. Jiao Z. Hsu L.-M. Zhou Z.. (2019). A deep learning framework for noise component detection from resting state functional MRI, in Medical Image Computing and Computer Assisted Intervention – MICCAI 2019, eds Shen D. Liu T. Peters T. M. Staib L. H. Essert C. Zhou S. Yap P.-T. Khan A. (Shenzhen: Springer International Publishing), 754–762.

[B17] Kohls G. Thönessen H. Bartley G. K. Grossheinrich N. Fink G. R. Herpertz-Dahlmann B.. (2014). Differentiating neural reward responsiveness in autism versus ADHD. Dev. Cogn. Neurosci. 10, 104–116. 10.1016/j.dcn.2014.08.003 25190643 PMC6987952

[B18] Li H. Chen Z. Gong Q. Jia Z. (2020). Voxel-wise meta-analysis of task-related brain activation abnormalities in major depressive disorder with suicide behavior. Brain Imaging Behav. 14, 1298–1308. 10.1007/s11682-019-00045-3 30790165

[B19] Li H. J. Hou X. H. Liu H. H. Yue C. L. He Y. Zuo X. N. (2015). Toward systems neuroscience in mild cognitive impairment and Alzheimer's disease: A meta-analysis of 75 fMRI studies. Hum. Brain Mapp. 36, 1217–1232. 10.1002/hbm.22689 25411150 PMC6869191

[B20] Lu Y. Ma Y. Chen C. Wang Y. (2018). Classification of single-channel EEG signals for epileptic seizures detection based on hybrid features. Technol Health Care 26, S337–S346. 10.3233/THC-174679 29710759 PMC6004942

[B21] Lunkova E. Guberman G. I. Ptito A. Saluja R. S. (2021). Noninvasive magnetic resonance imaging techniques in mild traumatic brain injury research and diagnosis. Hum. Brain Mapp. 42, 5477–5494. 10.1002/hbm.25630 34427960 PMC8519871

[B22] Luo F.-F. Wang J.-B. Yuan L.-X. Zhou Z.-W. Xu H. Ma S.-H.. (2020). Higher sensitivity and reproducibility of wavelet-based amplitude of resting-state fMRI. Front. Neurosci. 14, 224. 10.3389/fnins.2020.00224 32300288 PMC7145399

[B23] Mandelkow H. de Zwart J. A. Duyn J. H. (2017). Effects of spatial fMRI resolution on the classification of naturalistic movies. Neuroimage 162, 45–55. 10.1016/j.neuroimage.2017.08.053 28842385 PMC9881349

[B24] Mousavi Z. Yousefi Rezaii T. Sheykhivand S. Farzamnia A. Razavi S. N. (2019). Deep convolutional neural network for classification of sleep stages from single-channel EEG signals. J. Neurosci. Methods 324, 108312. 10.1016/j.jneumeth.2019.108312 31201824

[B25] Norman K. A. Polyn S. M. Detre G. J. Haxby J. V. (2006). Beyond mind-reading: multi-voxel pattern analysis of fMRI data. Trends Cogn. Sci. 10, 424–430. 10.1016/j.tics.2006.07.005 16899397

[B26] Pan P. Zhu L. Yu T. Shi H. Zhang B. Qin R.. (2017). Aberrant spontaneous low-frequency brain activity in amnestic mild cognitive impairment: a meta-analysis of resting-state fMRI studies. Ageing Res. Rev. 35, 12–21. 10.1016/j.arr.2016.12.001 28017880

[B27] Ren Y. Nguyen V. T. Sonkusare S. Lv J. Pang T. Guo L.. (2018). Effective connectivity of the anterior hippocampus predicts recollection confidence during natural memory retrieval. Nat. Commun. 9, 4875–4886 10.1038/s41467-018-07325-4 30451864 PMC6242820

[B28] Sasai S. Koike T. Sugawara S. K. Hamano Y. H. Sumiya M. Okazaki S.. (2021). Frequency-specific task modulation of human brain functional networks: a fast fMRI study. Neuroimage 224, 117375. 10.1016/j.neuroimage.2020.117375 32950690

[B29] Tzourio-Mazoyer N. Landeau B. Papathanassiou D. Crivello F. Etard O. Delcroix N.. (2002). Automated anatomical labeling of activations in SPM using a macroscopic anatomical parcellation of the MNI MRI single-subject brain. Neuroimage 15, 273–289. 10.1006/nimg.2001.0978 11771995

[B30] Übeyli E. D. (2009). Combined neural network model employing wavelet coefficients for EEG signals classification. Dig. Signal Proc. Rev. J. 19, 297–308. 10.1016/j.dsp.2008.07.004

[B31] Uddin L. Q. Dajani D. R. Voorhies W. Bednarz H. Kana R. K. (2017). Progress and roadblocks in the search for brain-based biomarkers of autism and attention-deficit/hyperactivity disorder. Transl. Psychiatry 7, e1218. 10.1038/tp.2017.164 28892073 PMC5611731

[B32] Wang J. Deng X. P. Wu Y. Y. Li X. L. Feng Z. J. Wang H. X.. (2020). High-frequency rTMS of the motor cortex modulates cerebellar and widespread activity as revealed by SVM. Front. Neurosci. 14, 1–12. 10.3389/fnins.2020.00186 32265624 PMC7096733

[B33] Wang J. Zuo X. Dai Z. Xia M. Zhao Z. Zhao X.. (2013). Disrupted functional brain connectome in individuals at risk for Alzheimer's disease. Biol. Psychiatry 73, 472–481. 10.1016/j.biopsych.2012.03.026 22537793

[B34] Wang X. Liang X. Jiang Z. Nguchu B. A. Zhou Y. Wang Y.. (2020). Decoding and mapping task states of the human brain via deep learning. Hum. Brain Mapp. 41, 1505–1519. 10.1002/hbm.24891 31816152 PMC7267978

[B35] Wang Z. Childress A. R. Wang J. Detre J. A. (2007). Support vector machine learning-based fMRI data group analysis. Neuroimage 36, 1139–1151. 10.1016/j.neuroimage.2007.03.072 17524674 PMC2717002

[B36] Wen H. Shi J. Zhang Y. Lu K. H. Cao J. Liu Z. (2018). Neural encoding and decoding with deep learning for dynamic natural vision. Cerebr. Cortex 28, 4136–4160. 10.1093/cercor/bhx268 29059288 PMC6215471

[B37] Zang Y. F. Yong H. Chao-Zhe Z. Qing-Jiu C. Man-Qiu S. Meng L.. (2007). Altered baseline brain activity in children with ADHD revealed by resting-state functional MRI. Brain Dev. 29, 83–91. 10.1016/j.braindev.2006.07.002 16919409

[B38] Zhang H. Gao Z. Z. Zang Y. F. (2015). An fMRI study of local synchronization in different subfrequency bands during the continuous feedback of finger force. Biomed. Res. Int. 8. 10.1155/2015/273126 26180789 PMC4477192

[B39] Zhang J. Yao R. Ge W. Gao J. (2020b). Orthogonal convolutional neural networks for automatic sleep stage classification based on single-channel EEG. Comput. Methods Programs Biomed. 183, 105089. 10.1016/j.cmpb.2019.105089 31586788

[B40] Zhang J. Zhang G. Li X. Wang P. Wang B. Liu B. (2020a). Decoding sound categories based on whole-brain functional connectivity patterns. Brain Imag. Behav. 14, 100–109. 10.1007/s11682-018-9976-z 30361945

[B41] Zhang X. Liu J. Yang Y. Zhao S. Guo L. Han J.. (2021). Test–retest reliability of dynamic functional connectivity in naturalistic paradigm functional magnetic resonance imaging. Hum. Brain Mapp. 2021, 1463–1476. 10.1002/hbm.25736 34870361 PMC8837589

[B42] Zhang Y. Zhang H. Chen X. Lee S. W. Shen D. (2017). Hybrid high-order functional connectivity networks using resting-state functional MRI for mild cognitive impairment diagnosis. Sci. Rep. 7, 1–16. 10.1038/s41598-017-06509-0 28747782 PMC5529469

[B43] Zhang Z. Telesford Q. K. Giusti C. Lim K. O. Bassett D. S. (2016). Choosing wavelet methods, filters, and lengths for functional brain network construction. PLoS ONE 11, 1–24. 10.1371/journal.pone.0157243 27355202 PMC4927172

[B44] Zhou Z. Wang J. B. Zang Y. F. Pan G. (2018). PAIR comparison between two within-group conditions of resting-state fMRI improves classification accuracy. Front. Neurosci. 11, 1–13. 10.3389/fnins.2017.00740 29375288 PMC5767225

[B45] Zimnik A. J. Lara A. H. Churchland M. M. (2019). Perturbation of macaque supplementary motor area produces context- independent changes in the probability of movement initiation Department of Neuroscience, Columbia University Medical Center, New York, New York, USA. Grossman Center for the Statisti. J. Neurosci. 39, 3217–3233. 10.1523/JNEUROSCI.2335-18.2019 30755488 PMC6788817

